# The replicometer is broken: telomeres activate cellular senescence in response to genotoxic stresses

**DOI:** 10.1111/acel.12246

**Published:** 2014-07-18

**Authors:** Anitha Suram, Utz Herbig

**Affiliations:** Department of Microbiology and Molecular Genetics, NJMS Cancer Center, Rm G1226 Rutgers Biomedical and Health Sciences205 South Orange Ave., Newark, NJ, 07103, USA

**Keywords:** aging, cellular senescence, DNA damage, hTERT, p53, telomere, telomerase, tumor suppression

## Abstract

Telomeres, the ends of our linear chromosomes, can function as ‘replicometers’, capable of counting cell division cycles as they progressively erode with every round of DNA replication. Once they are critically short, telomeres become dysfunctional and consequently activate a proliferative arrest called replicative senescence. For many years, telomeres were thought to be autonomous structures, largely isolated from cell intrinsic and extrinsic signals, whose function is to prevent limitless cellular proliferation, a characteristic of most cancer cells. It is becoming increasingly evident, however, that telomeres not only count cell divisions, but also function as sensors of genotoxic stresses to stop cell cycle progression prematurely and long before cells would have entered replicative senescence. This stable growth arrest, triggered by dysfunctional telomeres that are not necessarily critically short, likely evolved as a tumor-suppressing mechanism as it prevents proliferation of cells that are at risk for acquiring potentially hazardous and transforming mutations both *in vitro* and *in vivo*. Here, we review studies supporting the concept that telomeres are important cellular structures whose function not only is to count cell divisions, but also to act as molecular switches that can rapidly stop cell cycle progression permanently in response to a variety of stresses, including oncogenic signals.

## Introduction

Multicellular organisms with renewable tissues, such as humans, inevitably are at risk for developing diseases caused due to aberrant cell proliferation. The most common and deadly of these diseases is cancer. To counteract the development of cancer, these organisms developed a number of cell extrinsic and intrinsic tumor-suppressing mechanisms one of which is cellular senescence, a proliferative arrest that forces cells to exit the cell division cycle permanently. In cultured mammalian cells, senescence is activated in response to a number of signals including critical telomere erosion, oncogene activation, loss of tumor-suppressing pathways, as well as numerous cell extrinsic stresses (Campisi & d’Adda di Fagagna, 2007). Cells with features of senescence can also be detected in mammalian tissue suggesting that this stress response also arrests cellular proliferation *in vivo*.

Cellular senescence not only functions as a tumor-suppressing mechanism, but also has demonstrated roles in tissue repair (Rodier & Campisi, 2011) and embryonic development (Munoz-Espin *et al*., 2013). It therefore seems as if this stable proliferative arrest evolved to promote development and survival of the organism. Yet, not all aspects of the senescence response appear to benefit the organism. Senescent cells increase in abundance in a number of mammalian tissues in an aging-associated manner, thereby progressively depleting the organism of cells that might be critical for maintaining tissue structure and function. In addition, some senescent cells secrete molecules such as growth factors, pro-inflammatory cytokines, and proteases, among others that, although essential for efficient tissue repair, also damage the organism. These secreted molecules likely contribute to certain aging-associated disorder, as elimination of senescent cells from aging mouse tissues significantly improves animal healthspan (Baker *et al*., 2011). Furthermore, molecules secreted from senescent cells have the ability to facilitate cancer development, given that they stimulate proliferation of neighboring cancer cells both, *in vitro* and *in vivo* (Rodier & Campisi, 2011). Thus, the beneficial properties of cellular senescence are offset by a number of side effects, some which ultimately can be detrimental to the organism. Due to these opposing effects, cellular senescence has been proposed to be antagonistically pleiotropic, as it not only benefits the organism, but also damages it later in life (Rodier & Campisi, 2011).

Evidence that cellular senescence functions as a critical tumor-suppressing mechanism is strong, abundant and has been subject to a number of outstanding reviews in the past [for example see (Prieur & Peeper, 2008; Collado & Serrano, 2010)]. Using mouse model systems, various studies have demonstrated that oncogene expression leads to the formation of benign tumors in which cells display features of cellular senescence, such as high levels of certain heterochromatin proteins and senescence-associated-beta galactosidase (SA-βGal) activity. While neither SA-βGal activity nor all heterochromatin proteins characterize exclusively senescent cells, combining these markers with other features of cellular senescence, such as a lack of cellular proliferation markers among others, can more reliably identify senescent cells in tissue. Significantly, disruption of senescence pathways, for example by inactivating tumor suppressors p53 or INK4a, results in a loss of these senescence markers and promotes malignant cancer progression, supporting the notion that cellular senescence suppresses cancer development in mice. Cells with features of cellular senescence, such as high levels of certain heterochromatin proteins, SA-βGal activity, and lack of proliferation markers, also comprise certain benign human cancers and cancer precursor lesions, but are largely absent in the malignant cancer counterparts. These observations suggest that cellular senescence arrested cells in these lesions and prevented malignant cancer progression.

While abundant data now suggest that cellular senescence functions as a tumor-suppressing mechanism also in humans, it is less clear what caused cells to undergo this proliferative arrest in tissue. Frequently, signs of a persistent DNA damage response (DDR) can be detected in cells of inactive and benign human neoplasms, suggesting a role for DNA damage checkpoints in initiating and stabilizing this proliferative arrest. Recent data suggest that the DDR primarily initiates at telomeres, revealing a potentially critical role for dysfunctional telomeres in suppressing cancer growth in humans. In the following sections, we will review our current understanding of cellular senescence induced by a variety of stimuli and stresses that affect telomere length and structure, as well as summarize studies that shed light into the likely causes for telomere dysfunction and cellular senescence in cells of inactive and benign human tumors.

## Replicative senescence is an unlikely tumor-suppressing mechanism

Cellular senescence was first described in the early 1960’s by L. Hayflick, who observed that somatic human cells do not have the ability to divide indefinitely (Hayflick & Moorhead, 1961). He reported that cultured cells divided approximately 50 times before they underwent, what he termed, ‘senescence at the cellular level’ caused by an ‘accumulation of hits or errors in DNA replication which inactivated part of the genome’. Consistent with his hypothesis, it was proposed several years later that the protein complex replicating most of our DNA is not capable of completely duplicating the very ends of our linear chromosomes, the telomeres (Watson, 1972; Olovnikov, 1973). As a consequence of this end replication problem (and other factors; see below), telomeres progressively erode in cell cultures with every cell division cycle and eventually initiate a permanent proliferative arrest termed replicative senescence (RS). Because telomeres seem to shorten progressively and equally every time cells divide in culture and in tissues, they have been called ‘replicometers’ (Hayflick, 2000).

**Figure 1:**
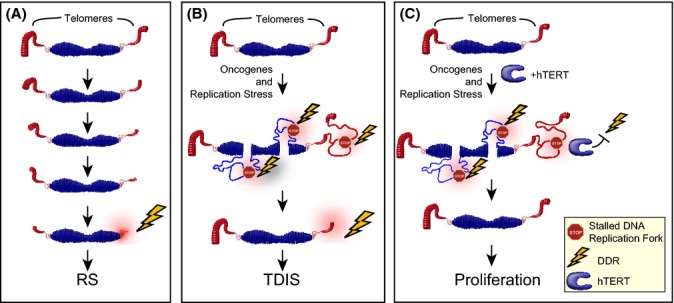
Telomerase protects cells from telomere dysfunction in response to DNA replication stress. (A) Progressive and moderate telomere erosion is observed following each cell division cycle. This is due to a combination of factors including the end replication problem, nucleolytic processing of chromosome ends, aberrant telomeric homologous recombination events, and oxidative damage, among others. Once telomeres are critically short, they initiate replicative senescence (RS) (B) Certain oncogenes cause hyperproliferation, DNA replication stress, and replication fork stalling, including in telomeric repeats. Some stalled replication forks are converted into double-strand DNA breaks, causing the activation of a DNA damage response (DDR). While most chromosome internal breaks are repaired, telomeric breaks impede repair activities, resulting in persistent telomeric DDR activation and telomere dysfunction-induced senescence (TDIS). (C) In the presence of high telomerase activity, DNA replication stress does not result in TDIS. This may be due to an ability of telomerase to suppress a telomeric DDR, facilitate replication of telomeric repeats, or promote repair of telomeric DNA breaks.

In humans, telomeres consist of 5–15 kb of repetitive TTAGGG sequences and end in a 100–200 nucleotide long G-rich overhang. Together with a number of protein factors, collectively called shelterin, telomeres form a looped structure to suppress the activation of a DDR at an otherwise exposed linear chromosome end. Progressive telomere shortening that is observed with every cell division cycle eventually leads to the formation of one or a few dysfunctional telomeres, a term used to describe telomeres that are sensed by the cell as double-stranded DNA breaks (Takai *et al*., 2003). A likely reason why some telomeres become dysfunctional following repeated cycles of cell divisions is that they had become so short that the protective telomere loop (t-loop) cannot be formed any longer. Consequently, the exposed chromosome end initiates a DDR that results in the activation of a persistent p53-dependent G1 DNA damage checkpoint (Campisi & d’Adda di Fagagna, 2007). This persistent checkpoint causes cells to undergo RS (Fig. 1A).

The problem that the mammalian replication machinery encounters at the telomere is that the primers used to initiate DNA synthesis are comprised of ~12 nucleotides of RNA which, at least in theory, are removed shortly after they primed synthesis of the last telomeric Okazaki fragment. The resulting loss of 12 telomeric nucleotides on the lagging strand would consequently result in an overall shortening of telomeric repeats following each round of DNA replication. Experimentally, however, a loss of 50–200 telomeric base pairs per cell division is observed in human cell cultures (Allsopp *et al*., 1992). These observations therefore suggest that either additional mechanisms contribute to telomere erosion in cultured cells or, alternatively, that the last RNA primer is synthesized somewhat internal to chromosome end. In fact, both scenarios appear to be encountered by somatic human cells. Factors that contribute to telomere erosion in mammalian cells include reactive oxygen species (ROS) that cause oxidative telomeric damage, telomeric secondary structures called G-quadruplexes (G4-DNA) that lead to replication problems within telomeres, as well as nucleolytic processing of chromosome ends, and homologous recombination events that result in sporadic telomere loss, among others (Lansdorp, 2005). Furthermore, recent data suggest that the RNA molecule used to prime the last telomeric Okazaki fragment is indeed located ~80 nucleotides internal to the very end of the chromosome and is removed approximately 1 h after its synthesis (Chow *et al*., 2012).

Why would such a tightly regulated and complicated replication machinery be so inefficient at replicating chromosome ends? A likely answer is that replicative polymerases do not have to duplicate the ends in their entirety. Instead, another complex specialized at maintaining telomeres performs this function in the great majority of eukaryotic organisms (Blackburn & Collins, 2011). Telomerase, a ribonucleoprotein complex consisting of a catalytic core subunit with reverse transcriptase activity (TERT), an associated RNA molecule (TERC or TR), and other less characterized protein components, is quite efficient in adding new telomeric repeats to the chromosome ends. Using the RNA component as an adapter to bind to the very 3′ end of a telomeric overhang, TERT subsequently synthesizes new telomeric repeats using TERC also as a template. Therefore, in cells that express sufficient levels of TERT, such as human stem and progenitor cells, telomere erosion is suppressed by the actions of telomerase. In contrast to these cells, however, somatic human cells lack high levels of telomerase and consequently lose telomeric repeats following each round of DNA replication both in culture and in tissue.

Following the discovery of the limited replicative potential of cells in culture, it was speculated that RS could function as a tumor-suppressing mechanism by preventing cells from dividing indefinitely. Arguing against this prediction, however, were observations that our cells have the ability to divide up to 80 times in culture, a number so great that the amount of cancer cells generated would kill the organism long before RS is initiated. Therefore, unless telomere erosion is accelerated in tissue compared to cultures, or telomeres can become dysfunctional regardless of their length, RS is an unlikely mechanism to stop growth of cancer cells.

## Oncogene-induced senescence is a tumor-suppressing mechanism

Cellular senescence can also be induced prematurely and long before telomere shortening, because continuous cell proliferation becomes growth limiting. While it is becoming increasingly evident that not all inducers of this premature growth arrest are entirely independent of telomeres (see below), cellular senescence can certainly be activated in the absence of any apparent telomere damage. Some cultured cell strains, for example, undergo cellular senescence without critical telomere erosion, visible DNA damage, or p53 activation. Instead, they initiate cellular senescence by upregulation of the Cdk inhibitor p16^INK4a^, which activates an Rb-dependent-senescence response. Given that the molecular activators and mediators of this growth arrest pathway are still poorly defined, it is currently not known to what extent it might function to suppress cancer growth in humans. However, highlighted by the fact that p16^INK4a^, the critical mediator of this senescence program, is inactivated in approximately one-third of human cancers, the importance of this telomere-independent proliferative arrest in preventing cancer initiation and progression cannot be underestimated (Romagosa *et al*., 2011).

Activating mutations in certain proto-oncogenes can also lead to a permanent proliferative arrest called oncogene-induced senescence (OIS). Depending on the cell type, strength, and duration of the oncogenic signal, oncogenes induce cellular senescence through multiple and distinct pathways (Courtois-Cox *et al*., 2008). Many cells upregulate p16 and p21 as a result of hyperproliferative signaling, suggesting that oncogenes signal through both the p53/p21 and the p16/Rb senescence pathways. For example, over-expression of activated H-RasV12 results in increased cellular ROS levels. ROS can cause cellular senescence either by causing DSBs, resulting in p21 upregulation, or by activating a kinase signaling cascade involving p38 and PRAK, which leads to p16 upregulation (Courtois-Cox *et al*., 2008). In cells that are more resistant to p16 upregulation, however, oncogenes have been demonstrated to promote DNA replication stress, a term used to describe problems during DNA synthesis that cause replication forks to slow down or stall. If DNA replication fails to resume at the stalled fork, as is the case in oncogene-expressing somatic cells, DSBs are generated in vicinity of the collapsed forks (Jossen & Bermejo, 2013). These DSBs can be detected as discrete DDR foci in cell nuclei by immunofluorescence microscopy using antibodies against DDR factors such as γH2AX or 53BP1 among others. Within few cell divisions, oncogene-expressing cells rapidly accumulate a large number of such DDR foci and consequently arrest the cell division cycle. While many of the oncogene-induced DDR foci are resolved, some persist and thus permanently activate DNA damage checkpoint that consequently engages the senescence response. Oncogenes that have been shown to cause DNA replication stress include constitutively active and/or overexpressed H-RasV12, BrafE600, Mos, Cdc6, Cyclin E, E2F1, and STAT5 (Gorgoulis & Halazonetis, 2010). Thus, while OIS is a term used to describe an irreversible growth arrest triggered by oncogenic signals, the pathways activated by oncogenes in cultured mammalian cells are diverse and cell-type-specific.

First direct evidence that OIS can function as a tumor-suppressing mechanism in mouse model systems was published almost a decade ago and has since been verified in numerous other studies (Collado & Serrano, 2010). Cells displaying features of cellular senescence have also been characterized in benign and premalignant human tumors, such as nevi, colon adenomas, prostate intra-epithelial neoplasias, and ductal breast hyperplasias, among others, but not in the malignant cancer counterparts, suggesting that cellular senescence suppresses progression of human cancers at premalignant stages (Collado & Serrano, 2010; Suram *et al*., 2012). Initiation of aberrant cell proliferation often is associated with oncogenic events in these lesions, suggesting that OIS indeed is an *in vivo* biological response of human cells encountering oncogenic signaling imbalances (Croce, 2008). With few exceptions, a common feature of the analyzed cancer precursor lesions is that cell nuclei within these benign tumors express markers of an activated DDR, which is consistent with the model that cells within these lesions had arrested as a result of oncogene-induced DNA replication stress (Gorgoulis & Halazonetis, 2010).

Precisely, how oncogenes cause DNA replication stress and DSBs is still not entirely clear. Cells expressing oncogenic H-Ras show signs of DNA hyper-replication, such as increased numbers of active replication origins as well as origins that appear to have initiated replication more than once per cell cycle (Di Micco *et al*., 2006). These cells also display a greater number of asymmetric and prematurely terminated replication forks compared to controls, which is also observed in cyclin E expressing cells (Bartkova *et al*., 2006), demonstrating that oncogene expression blocks DNA replication fork progression (Di Micco *et al*., 2006). While the barriers to fork movement in these cells have not yet been identified, it has been suggested that altered transcriptional profiles in oncogene-expressing cells could interfere with the processivity of the replication machinery and/or suppress deoxyribonucleotide biosynthesis, thereby depleting cells of components essential for DNA synthesis (Bester *et al*., 2011; Mannava *et al*., 2013). Another possibility is that the high levels of ROS found in oncogene-expressing cells damage DNA directly. Consequently, these oxidative lesions promote the recruitment of repair factors that potentially interfere with replication fork progression (Sedelnikova *et al*., 2010). What is evident, however, is that oncogene-induced replication stress leads to DSBs and other chromosomal aberrations preferentially in regions on the chromosomes that are prone to breakage and rearrangements, regions that are called common fragile sites (CFS) (Di Micco *et al*., 2006; Tsantoulis *et al*., 2008).

Common fragile sites are chromosomal loci that exhibit discontinuities, such as gaps and breaks, when viewed on metaphase chromosomes. Cells cultured under conditions that cause DNA replication stress, such as incubation with the DNA polymerase inhibitor aphidicolin, display an increased number of these chromosomal abnormalities, revealing that CFS are generated while cells replicate DNA. Functional disruption of kinases ATR and Chk1, which function to signal a stalled replication fork, as well as deficiency in recombination factors Rad51, BRCA1, and BLM, that act during the recovery of a stalled replication fork, enhances CFS expression, which is consistent with the model that CFS are generated as a result of DNA replication fork stalling. Significantly, CFS are not randomly distributed on chromosomes. Rather, they appear at a limited number of discrete loci indicating that specific features inherent to CFS sequences present a challenge to the replication machinery. Indeed, CFS often contain sequences that are prone to forming secondary structures and therefore act as a natural barrier to replication fork progression (Durkin & Glover, 2007).

## Telomeres pose a challenge to the DNA replication machinery

In recent years, it has become evident that CFS are much more abundant than previously thought. With the discovery that telomeres share many features of CFS, including the appearance of telomeric discontinuities on metaphase chromosomes following exposure to replication stresses (Martinez *et al*., 2009; Sfeir *et al*., 2009), as well as sensitivity to ATR (McNees *et al*., 2010; Pennarun *et al*., 2010), Rad51 (Badie *et al*., 2010), and BLM disruption (Sfeir *et al*., 2009), our chromosome ends can also be considered CFS. Previous studies that mapped location of aphidicolin-induced CFS likely did not detect structural abnormalities at telomeres, given that they are located at the extreme tips of our chromosomes. Only using fluorescently labeled probes that bind to the repetitive TTAGGG repeats was possible to detect discontinuities in telomeric chromatin compaction, apparent as multitelomeric signals, in response to DNA replication stresses (Martinez *et al*., 2009; Sfeir *et al*., 2009). Critical for facilitating telomere replication and suppressing the formation of fragile telomeres is TRF1, a component of shelterin that directly binds to double-stranded telomeric DNA. Also important for efficient telomere replication are BLM and RTEL1, as shRNA-mediated knockdown or conditional deletion of these helicases also leads to telomeric abnormalities, including a prominent fragile telomere phenotype (Vannier *et al*., 2012). Knockdown of these helicases in TRF1 deficient cells did not further increase the fragile telomere phenotype caused as a result of TRF1 ablation, suggesting that these factors act in concert to facilitate telomere replication (Sfeir *et al*., 2009). Significantly, interstitial telomeric repeats also cause aphidicolin-induced CFS, demonstrating that fragility is attributed to repetitive TTAGGG sequences and not to the terminal location of these repeats (Bosco & de Lange, 2012).

Why telomeres impede replication fork progression is still subject to investigation. Repetitive DNA sequences, in general, are known to challenge efficient and accurate DNA replication. This is primarily due to an increased abundance of hairpin secondary structures, due to polymerase slippage, or primer template misalignments (Voineagu *et al*., 2009). Another reason why telomeres might be particularly difficult to replicate is because of their propensity to form G4-DNA secondary structures. Indeed, molecules that stabilize G4-DNA promote the formation of fragile telomeres while helicases that resolve these secondary structures, such as BLM and RTEL1, facilitate replication of telomeric repeats (Sfeir *et al*., 2009; Vannier *et al*., 2012). Furthermore, one cannot exclude the possibility that increased telomeric DNA repair activities prevent the replicative polymerases from efficiently duplicating our chromosome ends. Due to their G-rich content, telomeric DNA is particularly susceptible to oxidative stress, which can result in the accumulation of telomeric single-stranded DNA nicks and 8-oxo-dG base damage (von Zglinicki *et al*., 2000). These lesions not only are less efficiently repaired in telomeres compared to other regions in the genome, but they also have been shown to reduce the binding of shelterin components TRF1 and TRF2 to chromatin. Consequently, oxidative stress reduces the efficiency of telomere replication (Opresko *et al*., 2005). Also, supporting this are studies demonstrating that deletion of factors involved in resolving oxidative base damage, such as Ogg1 and Nth1, results in telomeric abnormalities including fragile telomeres and increased telomeric DSB formation (Wang *et al*., 2010; Vallabhaneni *et al*., 2013). Thus, telomere replication is a process that is challenged by a multitude of factors in somatic human cells.

Failure to restart DNA synthesis at stalled replication forks, telomeric or nontelomeric, generates DSBs, and consequently DDR foci, near the stalled fork and causes cells to arrest the cell division cycle (Jossen & Bermejo, 2013). While our cells have developed a number of strategies to efficiently repair most chromosomal DSBs and thereby allow these to eventually continue proliferating, at least one region on our chromosomes, the telomere, resists these repair activities (Fumagalli *et al*., 2012; Hewitt *et al*., 2012). Cells exposed to certain genotoxic stresses, such as DNA replication stress, ionizing radiation, endonucleases, or ROS, rapidly acquire DSBs and activate a DDR following exposure. In mammalian cells with functional DNA damage responses, the great majority of DDR foci are resolved within minutes to hours of being generated, suggesting that most DSBs are repaired. In contrast, breaks that occur within telomeric repeats persist for at least several months, probably years, both *in vitro* and *in vivo* (Fumagalli *et al*., 2012; Hewitt *et al*., 2012). Persistent telomeric DDR foci are also generated in the presence of endogenous or ectopically expressed telomerase in response to ionizing radiation, suggesting that not even telomerase is able to facilitate repair of a telomeric break once it is generated. Even oncogenes, that for many years were thought to trigger cellular senescence independent of telomeres, were recently shown to cause transient nontelomeric and persistent telomeric DDR foci, suggesting that OIS is only stable because telomeric DSBs are irreparable (Suram *et al*., 2012). Importantly, whether they are generated as a result of progressive telomere erosion, DNA replication stress, telomeric DSB formation, or other genotoxic events, dysfunctional telomeres trigger cellular senescence in normal somatic cells. As it is often difficult to determine the causes of telomere dysfunction, we have called this proliferative arrest telomere dysfunction-induced senescence or TDIS (Fig. 1B).

## Telomere dysfunction-induced senescence is a tumor-suppressing mechanism

Why should telomeres be resistant to DNA repair activities? Linear DNA molecules are inherently unstable and either rapidly become degraded or fused to other exposed DNA ends by cellular DNA repair mechanisms to suppress DDR signaling. One critical function of our chromosome ends, therefore, is to suppress chromosome degradation and prevent end fusions with telomeres of sister chromatids, or with telomeres of other chromosomes. One mechanism that this could be accomplished is if telomeres, or associated proteins, somehow inhibit the DNA damage repair machinery. Indeed, components of shelterin, including TRF2, inhibit the functions of essential DSB checkpoint kinases as well as DSB repair factors and thereby suppress telomere end-to-end fusions (Sfeir, 2012). As a consequence of these inhibitory activities of telomere repeat-binding proteins, however, repair is not only suppressed at the very ends of our telomeres, but also at DSBs that occur within telomeric repeats.

At first thought, it might seem disadvantageous for the organism to prevent DSB repair in telomeric repeats. As our cells are frequently exposed to genotoxic stresses, occasional DSBs in telomeric sequences would force these cells to senesce prematurely, accumulate in our tissues, and potentially negatively impact tissue function as we age. Indeed, cells with persistent telomeric breaks, or dysfunctional telomeres, have been reported to increase in abundance in an aging-associated manner in tissue of mice (Hewitt *et al*., 2012), baboon (Herbig *et al*., 2006; Jeyapalan *et al*., 2007; Fumagalli *et al*., 2012), and humans (Suram *et al*., 2012). Yet, as repair of DSBs often is accomplished by mechanisms that cause mutations at the breakage site, providing the cell with a means to detect frequent genotoxic events would ultimately benefit the organism by inactivating cells that otherwise would have accumulated numerous mutations. In that sense, telomeres might function as sensors of genotoxic stresses that permanently stop cell cycle progression should these stresses become too abundant. The formation of irreparable telomeric DSBs, however, might serve an even more important purpose: that of protecting the organism from uncontrolled proliferation of cells harboring oncogenic mutations or encountering DNA replication stress due to hyperproliferation. Supporting this are data demonstrating that DNA replication stress, induced by oncogenic H-Ras, BRaf, or drugs, leads to the formation of transient nontelomeric and persistent telomeric DDR foci in somatic human cells. While both the nontelomeric and the telomeric DSBs likely cause a proliferative arrest, it seems that the persistent telomeric DSBs stabilize this arrest and force cells to undergo TDIS. The stability of this proliferative arrest is further increased by molecules that are secreted from senescent cells, including ROS that cause both telomeric and nontelomeric DSBs, in an autocrine and paracrine manner (Correia-Melo *et al*., 2014). Also, in cells of early neoplastic and benign human lesions, including nevi, colonic adenomas, and ductal breast hyperplasias, the persistent DDR foci are primarily telomeric, suggesting that cells within these lesions had become senescent due to the irreparability of telomeric DSBs (Suram *et al*., 2012). Whether induced due to gradual telomere erosion, oncogene-induced DNA replication stress, or other genotoxic stresses, current evidence therefore suggests that TDIS has evolved to protect us from developing malignant cancer.

While telomerase cannot prevent formation of telomeric DDR foci in response to ionizing radiation, or even resolve these foci with high efficiency once generated (Fumagalli *et al*., 2012; Hewitt *et al*., 2012), it can suppress the formation of a dysfunctional telomere under conditions that cause DNA replication stress (Fig. 1C). Supporting this are data demonstrating that neither drug-induced DNA replication stress nor expression of the oncogenes H-RasV12 or BRafV600 can induce formation of telomeric DDR foci in cells over-expressing catalytically active hTERT (Suram *et al*., 2012). These results therefore suggest that telomerase either promotes replication of telomeres, suppresses a telomeric DDR in S phase, or facilitates repair of a telomeric DSB generated at stalled replication forks. A role for hTERT during telomere replication in somatic human cells has indeed been suggested previously in a study demonstrating that hTERT levels and activity increase as cells progress through S phase (Masutomi *et al*., 2003). As telomerase cannot efficiently prevent replication fork stalling, or the formation of fragile telomeres in response to DNA replication stress (Sfeir *et al*., 2009; Suram *et al*., 2012), it is possible that it promotes repair of telomeric lesions generated as a result of replication fork stalling during S phase. One possible mechanism how telomerase suppresses activation of a telomeric DDR in response to DNA replication stress is that it adds *de novo* telomeric repeats to the exposed 3′ overhang of a telomeric DSB or single-stranded nick close to a stalled telomeric replication fork, thereby preventing telomere dysfunction. This mechanism, which resembles chromosome healing, has been described previously in cells that display telomerase activity (Murnane, 2010).

Because telomerase suppresses the formation of a telomeric DDR in response to DNA replication stress, cells expressing hTERT are significantly less sensitive to hyperproliferative signals and stresses that impede replication fork progression. While somatic human cells undergo TDIS prematurely and rapidly in response to DNA replication stress induced by drugs, oncogenes, or impairment of DNA replication factors, cells that over express hTERT, or cancer cells that have reactivated telomerase expression, are generally insensitive to these treatments (Suram *et al*., 2012) (and V. Boccardi, N. Razdan, U. Herbig, unpublished data). Significantly, also OIS induced by oncogenic H-Ras or BRaf over-expression is not stable in somatic human cells expressing high levels of hTERT as demonstrated recently (Kohsaka *et al*., 2011; Suram *et al*., 2012). Furthermore, these observations suggest that the reason why telomerase is reactivated in the great majority of malignant human cancers is not only to prevent telomere erosion, but also to suppress formation of telomeric DSBs that would otherwise be generated due to hyperproliferation and DNA replication stress. They also highlight that TDIS likely only suppresses cellular proliferation at early stages during cancer development and in cells with low telomerase activity, as cells in malignant human cancers with high telomerase activity would be largely insensitive to oncogene-induced telomeric replication stress. These new data additionally have important implications for therapeutic strategies that target and inhibit telomerase activity in more advanced human cancers, given that initial attempts to combat various cancer types using telomerase inhibitors have suffered some setbacks (Williams, 2013). In addition, one concern about the efficacy of telomerase inhibitors is that, despite immediate inhibition of telomerase, they would still allow extensive cancer cell proliferation until telomeres have eroded to such an extent that would cause the cells to undergo senescence or apoptosis. If, however, these cancer cells are encountering high levels of DNA replication stress, due to oncogene expression, other hyperproliferative signals, or drugs, telomerase inhibitors potentially could arrest cancer cell proliferation much more rapidly.

## Conclusions and perspectives

It is becoming increasingly evident that telomeres are far from being replicometers that simply count cell division cycles. Mounting evidence supports the model that telomeres also function as sensors of genotoxic events, capable of shutting down cell cycle progression prematurely before telomeres had eroded to critically short lengths. Furthermore, while telomere dysfunction can promote cancer progression by seeding the events that lead to genomic instability in cells with compromised DNA damage responses, it is also clearly beneficial for the organism as it promotes cell death and stabilizes cellular senescence as demonstrated both in animal model systems (Cosme-Blanco *et al*., 2007; Feldser & Greider, 2007) and in humans (Suram *et al*., 2012). The tumor-suppressing properties of TDIS depends on functional DNA damage checkpoints and therefore likely act early, during the initial stages of cancer development.

Cells with features of TDIS have been detected in inactive and benign lesions in the skin, as well as in colonic and breast epithelium. TDIS therefore can be activated in distinct cell types and throughout our tissues. While we don’t yet have a thorough knowledge of what other tissues are protected from developing neoplasms due to activation of TDIS, future studies should reveal the entire extent to which this tumor-suppressing mechanism protects us from developing malignant cancers. Also still unresolved is how telomerase suppresses formation of dysfunctional telomeres and induction of TDIS in response to DNA replication stress. As this function requires its catalytic activity, it is also targetable using existing antitelomerase drugs which were designed to inhibit the telomere extending abilities of telomerase. The vulnerability of telomeres to oncogenic and hyperproliferative signals in the absence of telomerase activity could therefore be exploited by using drug combination cancer therapies, targeting both telomerase while inducing DNA replication stress. While much still remains to be learned about the role of dysfunctional telomeres during aging and cancer development, it is becoming increasingly evident that the beneficial and tumor-suppressing properties of telomere dysfunction are vital for human health.
